# Spectral and Energy Efficient Low-Overhead Uplink and Downlink Channel Estimation for 5G Massive MIMO Systems

**DOI:** 10.3390/e20020092

**Published:** 2018-01-30

**Authors:** Imran Khan, Mohammad Haseeb Zafar, Mohammad Tariq Jan, Jaime Lloret, Mohammed Basheri, Dhananjay Singh

**Affiliations:** 1 Department of Electrical Engineering, University of Engineering and Technology, Peshawar 814, Pakistan; 2 Department of Physics, Kohat University of Science and Technology (KUST), Kohat 26000, Pakistan; 3 Instituto de Investigación para la Gestión Integrada de Zonas Costeras, Universitat Politècnica de València, 46022 Camino de Vera, Spain; 4 Department of Information Technology, Faculty of Computing and Information Technology, King Abdulaziz University, Jeddah 21589, Saudi Arabia; 5 Department of Electronics Engineering, Hankuk University of Foreign Studies, Yongin 449-791, Korea

**Keywords:** 5G, CS, sparsity, feedback, pilot

## Abstract

Uplink and Downlink channel estimation in massive Multiple Input Multiple Output (MIMO) systems is an intricate issue because of the increasing channel matrix dimensions. The channel feedback overhead using traditional codebook schemes is very large, which consumes more bandwidth and decreases the overall system efficiency. The purpose of this paper is to decrease the channel estimation overhead by taking the advantage of sparse attributes and also to optimize the Energy Efficiency (EE) of the system. To cope with this issue, we propose a novel approach by using Compressed-Sensing (CS), Block Iterative-Support-Detection (Block-ISD), Angle-of-Departure (AoD) and Structured Compressive Sampling Matching Pursuit (S-CoSaMP) algorithms to reduce the channel estimation overhead and compare them with the traditional algorithms. The CS uses temporal-correlation of time-varying channels to produce Differential-Channel Impulse Response (DCIR) among two CIRs that are adjacent in time-slots. DCIR has greater sparsity than the conventional CIRs as it can be easily compressed. The Block-ISD uses spatial-correlation of the channels to obtain the block-sparsity which results in lower pilot-overhead. AoD quantizes the channels whose path-AoDs variation is slower than path-gains and such information is utilized for reducing the overhead. S-CoSaMP deploys structured-sparsity to obtain reliable Channel-State-Information (CSI). MATLAB simulation results show that the proposed CS based algorithms reduce the feedback and pilot-overhead by a significant percentage and also improve the system capacity as compared with the traditional algorithms. Moreover, the EE level increases with increasing Base Station (BS) density, UE density and lowering hardware impairments level.

## 1. Introduction

Massive MIMO is an upcoming technology for the future 5G Wireless Communication Systems [1]. It is a physical layer (PHY-L) envisioned technology that will resolve various physical layer issues in modern cellular networks. It has attracted substantial research interest from various communities for assessing its various merits. It has the ability to provide improved Spectral Efficiency (SE) and Energy Efficiency (EE) by utilizing the space-resources. It is considered to be the future enabler of bandwidth-efficient, energy-efficient, secure and robust broadband networks. It will be deployed on the Internet of Things (IoT), Smart City and many other emerging infrastructures such as e-health [2]. It has high spatial-resolution, simple hardware-design, and less inter-cell interference. It uses hundreds or even thousands of low-power, physically-compact, and independently controllable antennas at the Base Stations (BS) in order to serve multiple-users Mobile Stations (MSs) or User Equipment (UE) simultaneously using the same time-frequency resources [3]. It allows adding enhancements such as congestion control systems [4] and intelligent handover algorithms [5]. The data rate capacity is increased due to the spatial-multiplexing which transmit data to the desired UE. The energy efficiency is boosted due to the sharp and more focused beamforms for the desired user location [6].

Massive MIMO provides a favorable propagation channel thanks to a large number of deployed antennas. Such a channel has the important property that the channel-vectors and UEs become orthogonal in pairs, and this can be easily processed using simple linear signal processing techniques such as Matched Filtering (MF). Massive MIMO can also be deployed together with mmWave to efficiently utilize its bandwidth which is also a key technology in 5G Ultra-Dense Networks (UDNs) [7].

To obtain both SE and EE, it requires accurate Channel State Information (CSI) at the Transmitter (Tx) Side [8]. The allocated channel resources are limited and the Massive MIMO channel matrix size grows immensely; therefore, channel feedback at the Uplink (UL) turns out to be contesting problem. The feedback and the data signals are transmitted together, so if the feedback signal overhead is large then it will consume extra bits from the scarce spectrum, which is the problem that is addressed in this work. The conventional codebook channel feedback technique is not appropriate as its size should be greatly extended to ensure adequate CSI accuracy. The non-codebook channel estimation methods have high feedback overhead. Therefore, Compressive-Sensing (CS) and AoD techniques are employed here to decrease its overhead. For Downlink (DL) channel estimation, accurate CSI is also mandatory for improved SE and EE. The conventional DL channel estimation techniques such as Minimum Mean Square Error (MMSE) and Least Square (LS) are not appropriate because, the number of orthogonal-pilots increases directly with the number of BS antennas, and it causes excessively large-overhead. To decrease such overhead, we deploy an enhanced Block-ISD and S-CoSaMP algorithms. 

In this paper, we propose a novel approach of deploying various renowned CS-based algorithms for the efficient UL and DL channels estimation with low-overhead. We also analyze the EE of the system in terms of BS density, UE density and level of hardware impairment by deploying small cells in dense network scenario with the specific constraints. We deploy Compressed Sensing (CS), Block-ISD, AoD and S-CoSaMP algorithms to reduce the channel feedback and pilot overhead for UL and DL scenarios. Theoretical formulations are also provided for the better understanding and comparison. Such methods significantly decrease the channel overheads, while enhances the system performance. CS is a new signal-processing concept that can recover high-scale sparse-signals from low-scale measurements with an adequate probability [9]. The wireless channels have limited number of significant-paths which exhibit sparse response and that is considered as the basic principle by CS techniques. The Block-ISD fully utilizes the sparsity inside the block-sparse-equivalent Channel Impulse Response (CIR) which is obtained from the spatial-correlations of the considered CIRs. It is a better approach because it does not depend on prior knowledge of sparsity-level and it has more probability of practical implementations as compared to other schemes.

The AoD adaptive-subspace codebook utilizes the channel characteristics that the variation in path-AoDs is much slower than the variations in path-gains. It uses the phenomenon of channel-quantization to quantize the channel-vectors. The path-AoDs remains constant within the angle-coherence time. In the N-dimensional space, where N is the number of BS-antennas, the channel-vector is only distributed in the sub-space of it. It is also considered as the channel-subspace. The quantization-vectors are focused and mapped accurately on the normalized-channel subspace whose size is very smaller than the complete space. Thus, the number of feedback overhead bits is linearly proportional to the number of path-AoDs rather than the whole number of BS antennas, at that is the basis for reduced overhead. It also assists in keeping a constant data-rate gap without affecting the other parameters. Furthermore, our AoD codebook method is better than the conventional channel-statistics based method as it can track the channel-vectors more efficient. The S-CoSaMP scheme also provides a clue to reduce the channel overhead efficiently. Dense antenna-arrays provides more correlated signals at the BS. As the BS-antennas are closely-spaced which causes same path-delays, so the CIRs of each antenna has a common support vector which is deployed by S-CoSaMP to decrease the undesired overhead. S-CoSaMP recovers the CIR from the reduced pilots. This technique can further decrease the UL/DL feedback overhead. This scheme uses SCS-joint UL/DL mechanism for channel estimation. It also eliminates the need for channel estimation at each UE and then feedback to BS. UEs directly feedback their pilots to BS where CS-algorithms recover the corresponding CIR. It uses a joint mechanism which improves the system performance as compared to the conventional CS-algorithms that performs channel training and feedback separately.

The rest of the paper is organized as follows: Section 2 describes the literature review of the paper which discusses the related background work. Section 3 discusses the proposed system model with all necessary theoretical as well as mathematical explanation. Section 4 provides the simulation results which show a comparison between the proposed and the conventional algorithms in terms of various parameters. Finally, Section 5 provides our conclusions and future work.

*Notations:* Lowercase and upper-case letters denote vectors and matrices, respectively; ${\left(\cdot \right)}^{T},\;{\left(\cdot \right)}^{H}$, and ${\left(\cdot \right)}^{-1}$ denote the transpose, conjugate transpose, and inverse of a matrix, respectively; ϕ represents the sensing matrix, γ denotes the feedback overhead, $I_{K}$ denotes the identity matrix of K × K dimension; ρ is the correlation coefficient, $\alpha_{h_{i}}$ is the support element of CIR $h_{i}$ and µ is the channel sparsity.

## 2. Literature Review

The recent research activities indicate that the main goals of the 5G mobile communications networks are to accomplish 1000× times the system-capacity and 10× times the Spectral Efficiency (SE), Energy Efficiency (EE) and data-rate, and 25× times the average-cell-throughput [9]. The demand for high data-rate and link-reliability is increasing exponentially due to the increasing number of new generation of devices such as smart-phones, tablets and notebooks, etc. The electromagnetic spectrum is a precious but limited commodity, so, to utilize such spectrum efficiently and to cope with the demands, the high-level perspective technology of massive MIMO is deployed. Massive MIMO is defined as a system that deploys a large number of smaller array antennas at the BS which significantly improve the beamforming and system capacity and can simultaneously provide service to a large number of users (UEs) [10]. It has various merits over the conventional MIMO. First, it uses a large number of antennas at the BS due to which the simplest coherent-combiner and linear-precoder can be used for signal processing such MF or ZF. Second, by increasing the number of antennas increases the system capacity substantially using the channel-reciprocity features and without increasing feedback-overhead. Third, the reduced power benefits in the UL/DL provide the feasibility to shrink the cell-size which can be used in micro and pico-cells. Massive MIMO Technology is used by various wireless communication standards and companies such as the Long-Term-Evolution-Advanced (LTE-A), due to its substantial potential both in channel-capacity and route-reliability. It uses hundreds of antennas at the BS in various geometrical shapes in the form of arrays. Increasing the number of antennas assist to direct the radiated energy towards much smaller-regions, which improves the throughput and EE. It also provides aggressive-spatial multiplexing. It has the ability to decrease the transmitter (Tx) power and also enhances the Spectrum Efficiency (SE) by 10 times and more. Also, low-power devices are deployed for service purpose, which is also a plus point for massive MIMO as it is low-cost and easily manufactured from solid-state devices.

The performance characteristics of the wireless system are not solely based on the transmitter (Tx) and receiver (Rx), but are also dependent upon the wireless channel under consideration. The wireless channel is very complicated and unpredictable. It is the key element to understand the development of high-performance, improved Quality-of-Service (QoS) and bandwidth-efficient wireless communication technology. In Massive MIMO Systems, accurate UL and DL CSI are necessary for channel estimation [5]. In [3], it is illustrated that in propagation-environments, the MIMO-channels shows a sparse-structure in Channel Impulse Response (CIR). To reap the advantages of massive MIMO, accurate CSI is mandatory for each Tx-Rx pair. As there is a large number of antennas used by massive MIMO, therefore the channels estimations associated with such antennas will also be large which is a tedious task because of the enormous increasing dimensions of the channel matrix. Many channel estimation techniques for CIR are proposed in the related research literature which can be classified into superimposed [11], training [12], pilot [13], blind [14], and semi-blind [15] techniques, respectively.

The CS paradigm is getting much research attention as it has the ability to recover the unknown signals from just a small number of measurements. It uses very few samples following the Nyquist sampling rate. It deploys the non-zero elements of a sparse-signal for the recovery operation. It also performs accurate estimation of system-parameters with fewer-pilots, therefore it also increases the bandwidth (BW) efficiency. The classical conventional CS-paradigms need pre-known information of channel-sparsity, which is practically unknown in system analysis. Furthermore, for reliable and accurate CIR retrieving, the CS-sensing or sampling matrix must satisfy the isometry property.

The previous research performed for channel estimation did not perform UL/DL channel estimation jointly using CS [16,17,18,19], differential CIR [20], Block-ISD [21], AoD [22] and S-CoSaMP [23]. Therefore, we propose an efficient, low-overhead UL/DL joint channel feedback and pilot-based channel estimation which gives an optimum comparison with the conventional schemes such as direct-CS, classical Block-ISD, BP, LS, CoSaMP, Kalman filter, J-OMP and conventional codebook mechanisms respectively. The simulation results provide a clear understanding of the improvement caused by the proposed algorithms in terms of the mean square error (MSE), data-rate, per-user rate and feedback bits.

## 3. System Model

The proposed system is modeled using N_t_ transmitter antennas, *K* receiving antennas such that (N_t_ >> *K*), SNR, channel length, channel sparsity, slot-interval, and various relevant parameters to obtain the required results. 

### 3.1. CS-Differential Channel Feedback

**Figure 1:**
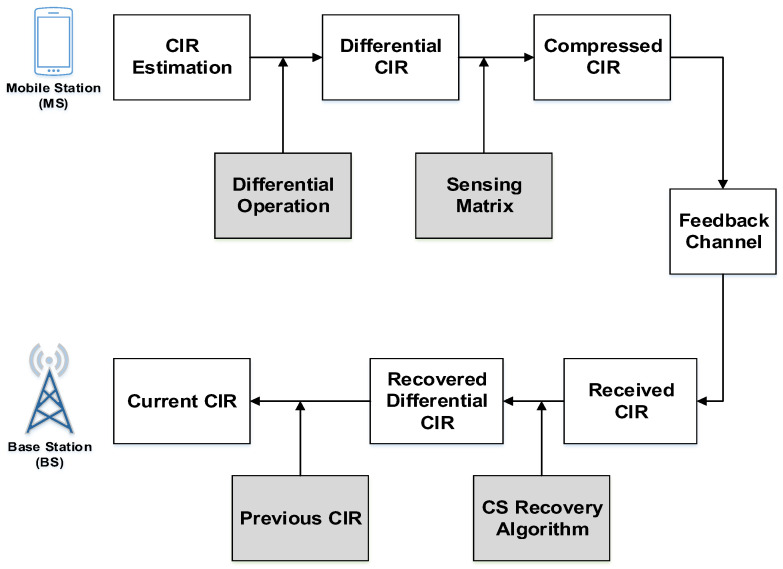
Proposed CS-differential UL channel-feedback.

The proposed CS algorithm for Uplink (UL) differential channel feedback is depicted in Figure 1. The CIR channel feedback is performed at particular time-slots. The direct compressed sensing (DCS) method does not perform the differential operation and directly compresses the CIR with sensing-matrix. But the CS differential algorithm applies differential operation for generating more strong and sparse Differential CIR (DCIR) than the DCS CIR. DCIR is then compressed to Compressed CIR (CCIR) using the sensing-matrix and it is known to base-station (BS) and User (UE). Such CCIR is fed back to BS. The CS Recovery algorithm gets back the DCIR from the CCIR accurately.

#### 3.1.1. Temporal-Correlation of Massive MIMO Channels

Consider k-time slots for the time-varying channels between *n*-number of BS antennas and single UE antenna. Mathematically, the Channel Impulse Response (CIR) at kth time-slot between the *n*th BS antenna and single UE is represented by:

(1) $$h_{n}^{\left(k\right)}={\left[h_{n}^{\left(k\right)}\left(0\right),\;h_{n}^{\left(k\right)}\left(1\right),\;h_{n}^{\left(k\right)}\left(2\right),\;\ldots ,\;h_{n}^{\left(k\right)}\left(L-1\right)\right]}^{T}\;\mathrm{for}\;1\leq n\leq N$$

where: *N*: Number of BS-antennas and L: Maximum channel Length.

The Sparse CIR is represented by a supportive-vector $p_{n}^{\left(k\right)}$ and an amplitude-vector $a_{n}^{\left(k\right)}$:

(2) $$h_{n}^{\left(k\right)}=p_{n}^{\left(k\right)}o\;a_{n}^{\left(k\right)}$$

where $p_{n}^{\left(k\right)}\left(l\right)\;∊$ {0, 1}, $a_{n}^{\left(k\right)}\;∊\;\mathbb{C}$ and “o” indicates the Hadamard-product.

#### 3.1.2. Support-Vector

$p_{n}^{\left(k\right)}$ having *l*th elements and T-vectors can be represented by a first-order Markov process that represent it by two transition-probabilities:

(3) $$p_{10}\;≜P_{r}\{p_{n}^{\left(k+1\right)}\left(l\right)=1|p_{n}^{\left(k\right)}\left(l\right)=0\}$$

and:

(4) $$p_{01}≜P_{r}\{p_{n}^{\left(k+1\right)}\left(l\right)=0|p_{n}^{\left(k\right)}\left(l\right)=1\}$$

where, $p_{10}$ is the transition probability from 0 (at time slot *k*) to 1 (at time slot *k* + 1). The corresponding distribution for initial time-slot (*k* = 1) is:

(5) $${µ}_{n}^{\left(1\right)}≜P_{r}\left\{p_{n}^{\left(1\right)}\left(l\right)=1\right\}$$

For the steady-state Markov process:

(6) $$P_{r}\{p_{n}^{\left(k\right)}\left(l\right)=1\}=µ$$

For all k, n. Where, µ denotes the channel sparsity. From the above equations, only $p_{01}$ and µ are important to represent the Markov process as $p_{10}$ can be obtained from $p_{01}$ and µ respectively.

#### 3.1.3. Amplitude-Vector

It is represented by a first-order auto-regressive process as [24]:

(7) $$a_{n}^{\left(k\right)}=\rho \;a_{n}^{\left(k-1\right)}+\;\sqrt{1-\;\rho^{2}}\omega^{\left(k\right)}$$

where $\omega^{\left(k\right)}$: is the noise-vector whose elements are independent identically distributed (i.i.d) with zero mean Gaussian variables which follow $\mathbb{C}N\left(0,\;\sigma_{\omega }^{2}\right)$ distribution and does not depend on $a_{n}^{\left(k-1\right)}$, ρ: is the correlation-coefficient which is obtained by the Bessel-function of zero order as follows:

(8) $$\rho =\;J_{o}\left(2\pi f_{d}\tau \right)$$

$f_{d}:$ Maximal Doppler-frequency and τ : Time-interval of slot.

#### 3.1.4. Differential CIR (DCIR)

It is determined by taking the difference among two CIRs at the previous at current time-slots, which is the required sparse-signal. Mathematically:

(9) $$\Delta h_{n}^{\left(k\right)}=\;h_{n}^{\left(k\right)}-h_{n}^{\left(k-1\right)}$$

By putting the corresponding values from (2) and (7) in (9) we get:

(10) $$=p_{n}^{\left(k\right)}o\;(a_{n}^{\left(k\right)}-a_{n}^{\left(k-1\right)})+(p_{n}^{\left(k\right)}-p_{n}^{\left(k-1\right)})\;o\;a_{n}^{\left(k-1\right)}\;=\;p_{n}^{\left(k\right)}o\left[\sqrt{1-\;\rho^{2}}\omega -\left(1-\rho \right)a_{n}^{\left(k-1\right)}\right]+(p_{n}^{\left(k\right)}-p_{n}^{\left(k-1\right)})o\;a_{n}^{\left(k-1\right)}$$

**Figure 2:**
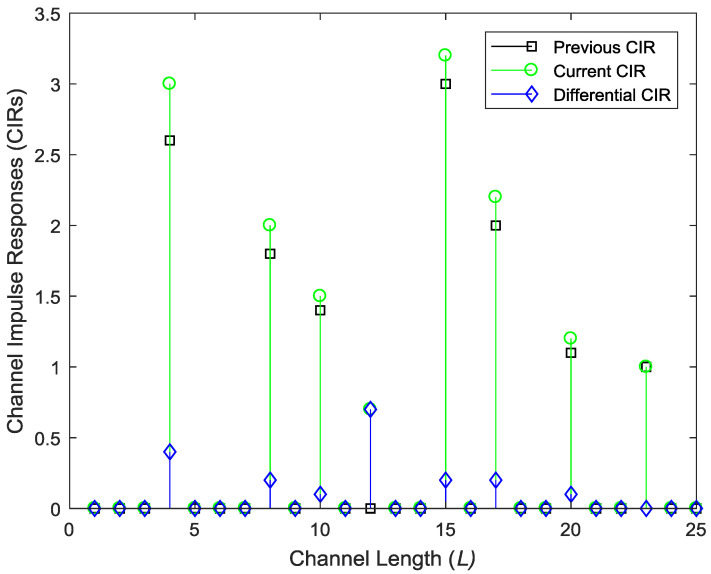
Illustration of the previous, current and differential channel impulse responses (CIRs) in different time slots with same channel length.

The above process is graphically illustrated in Figure 2, in which the previous CIR, current CIR and the differential CIR (DCIR) are shown. It is clear from the figure that the DCIR has more sparsity and can be more compressed than the other two CIRs.

#### 3.1.5. Compression CIR (CCIR)

The $\Delta h_{n}^{\left(k\right)}$ is compressed by the sensing matrix $\phi \;∊\;{\mathbb{C}}^{MxL}$ with M≪L and convert it into a lower level measurement-vector as follows:

(11) $$y=\phi \Delta h_{n}^{\left(k\right)}$$

y: compressed channel-feedback to BS.

#### 3.1.6. Feedback-Overhead

It is expressed as a ratio of channel compression w.r.t channel length. This ratio should be small enough for efficient channel-feedback and recovery process. Mathematically:

(12) $$\gamma =\frac{M}{L}$$

where M≪L so the ratio is less than 1 (100%) and it is added as extra overhead bits to the feedback channel attributes.

#### 3.1.7. CS-Recovery Algorithm

The desired $\Delta h_{n}^{\left(k\right)}$ is recovered by the BS from the noise accumulated signal if ϕ fulfills the isometric-property [25]. Mathematically:

(13) $$y=\phi \Delta h_{n}^{\left(k\right)}+n$$

where *n* represents the feedback-channel noise having the same attributes as the noise-vector ω in (7) and follows $\mathbb{C}N\left(0,\;\sigma_{n}^{2}\right)$.

#### 3.1.8. Flowchart

**Figure 3:**
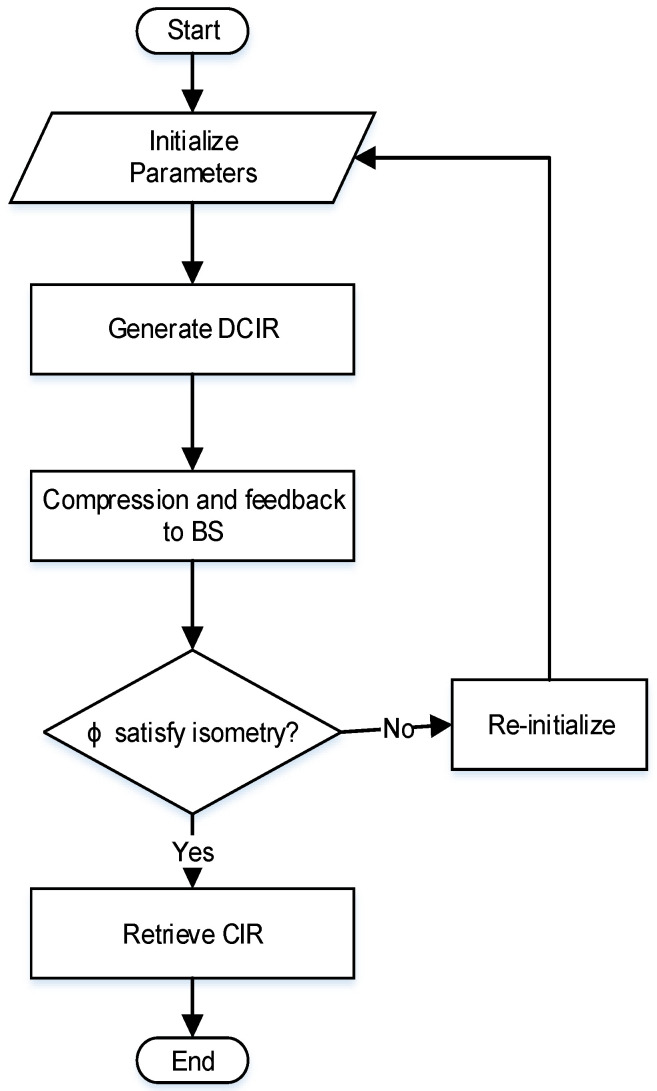
CS-UL channel-feedback flowchart.

The flowchart in Figure 3 describes the major steps involved in the CS-differential channel estimation and feedback process. First, the system parameters are initialized which are mentioned in Table 1. Then the differential operation is performed to produce DCIR. The sensing matrix then performs compressing operation for CCIR and that is feedback to BS for recovering. The condition checks whether φ obey isometry, if yes then the required CIR is obtained to complete the desired channel-estimation process.

### 3.2. Block-ISD Pilot Feedback

**Figure 4:**
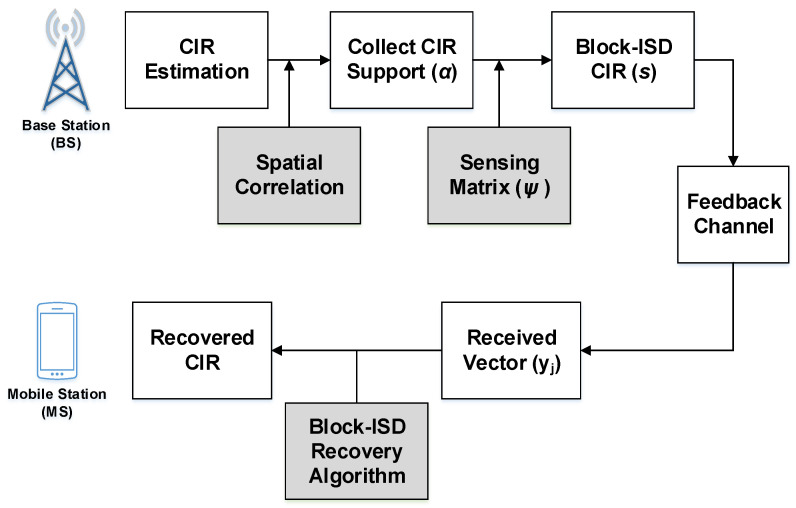
Proposed Block-ISD channel-feedback.

Figure 4 shows the proposed block-ISD based channel-feedback schemes. The same number of antennas are deployed for downlink channel estimation as in uplink channel estimation process. Orthogonal Frequency Division Multiplexing (OFDM) scheme is employed to determine the number of pilots easily in frequency-domain [26,27,28]. Mathematically, the received pilots-signals at the MS or UE is expressed as:

(14) $$y_{j}=\;\overset{N_{T}}{\underset{i=1}{\sum }}P_{i}{\left(F_{L}\right)}_{j}h_{i}+n_{j}\;$$

where j=[1,2, …, N]: Is the index of subcarrier-set that are chosen randomly and allocate to pilots-signals, $y_{j}$: Measurements performed for CIR recovery; $P_{i}$ = diag$\left(p_{i}\right)$: Is the diagonal matrix of the pilot-vector $p_{i}$ at the corresponding *i*th Tx antenna, $p_{i\;}∊\;{\mathbb{C}}^{px1}$ here *p* represents the number of pilot-signals; $F_{L}\;∊\;{\mathbb{C}}^{MxL}$ is the sub-matrix which contains the Discrete Fourier Transform (DFT) column matrix of L columns of order M × M; M is the symbol-length of OFDM; ${\left(F_{L}\right)}_{j}$ is the sub-matrix containing rows of $F_{L}$ sub-matrix with index of *j*; $h_{i}={\left[h_{i}\left(1\right),\;h_{i}\left(2\right),\;\ldots ,\;h_{i}\left(L\right)\right]}^{T}$ is the CIR between the *i*th BS antenna and single-user Rx antenna; *L* is the maximum channel-length; $n_{j}={\left[n_{1},\;n_{2},\;\ldots ,\;n_{p}\right]}^{T}$ is the channel noise-vector, having the same characteristics as discussed in Equation (13).

For the sake of convenience, (14) can also be written as:

(15) $$y_{j}=\;\phi \times h+n_{j}$$

where ϕ $=[P_{1}{\left(F_{L}\right)}_{j},\;P_{2}{\left(F_{L}\right)}_{j},\;\ldots ,\;P_{N_{T}}{\left(F_{L}\right)}_{j}$] is the sensing-matrix and $h={\left[h_{1}^{\left(T\right)},\;h_{2}^{\left(T\right)},\;\ldots ,\;\;h_{N_{T}}^{\left(T\right)}\right]}^{T}$ is the cumulative CIR from the $N_{T}$ number of antennas.

#### Creating Block-ISD CIR

For the recovery process, the CIR (*h*) is required which is extracted from the noise-accumulated signal. The spatial-correlation operation is performed on various CIRs of the considered antennas. The CIRs between the BS and UE of each antenna exhibit analogous properties such as the Time of Arrival (ToA), so they share a common-supporting element as follows [29]:

(16) $$\alpha_{h_{1}}=\alpha_{h_{2}}=\ldots =\;\alpha_{h_{N_{T}}}$$

where $\alpha_{h_{i}}=\left\{q\;:h_{i}\left(q\right)\neq 0\right\}$ is the support of $h_{i}$.

**Figure 5:**
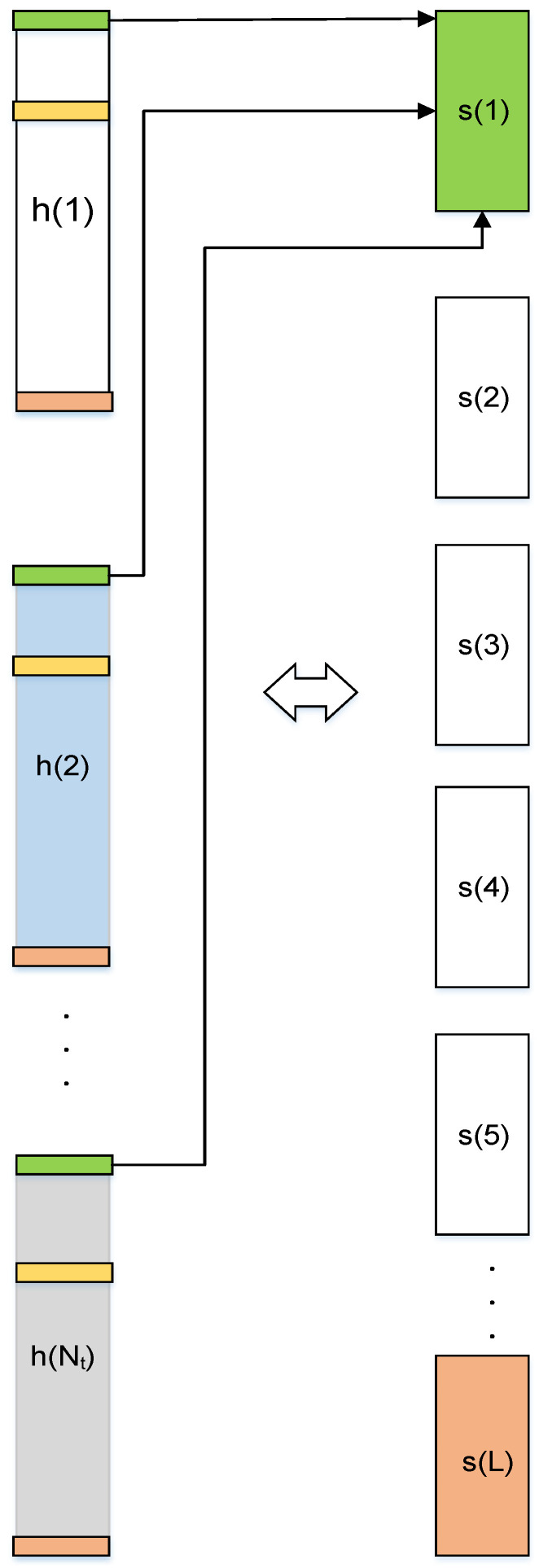
Creation of block-sparse equivalent-CIR.

Figure 5 illustrates the production of block-sparse equivalent CIR (*s*) from the considered CIRs which has more sparsity. The green, yellow and orange colors of CIRs (*h*) represents the non-zero elements and that is also the common support elements among them from which the desired sparse-CIR is constructed.

The equivalent CIR (*s*) is given as:

(17) $$s={\left[s_{1},\;s_{2},\;\ldots ,\;s_{L}\right]}^{T}$$

which is obtained from the following equation:

(18) $$s\left(\left(l-1\right)N_{T}\right)+n_{t})=h\left(\left(n_{t}-1\right)L+l\right)$$

where l=1, 2, …, L; $n_{t\;}=1,\;2,\;\ldots ,\;N_{T}$.

From (18), the zero and non-zero elements can be grouped separately for feasibility if s is decomposed into L-blocks where each block contains $N_{T}$-elements. Therefore, the supportive elements of s (i.e., $\alpha_{s}$) can be updated collectively using the basic pursuit (BP) technique. To represent the new sensing-matrix ψ with more-sparse attributes in terms of ϕ in (15) and (18) it is written as:

(19) $$\psi \left(:,\;\left(l-1\right)N_{T}\right)+\;n_{t})=\phi (:,\;\left(n_{t}-1\right)L+l)$$

and the corresponding estimated channel from (15) can be written as:

(20) $$y_{j}=\;\psi \times s+n_{j}$$

where $y_{j}$ Is the received vector of order *p* × 1; *s* is the require sparse CIR with the order $N_{T}L\times 1$.

### 3.3. AoD-Adaptive Subspace Codebook Algorithm

We consider the same number of BS and UE antennas as in CS and Block-ISD techniques. It is also based on CS algorithms. To obtain the DL channel-vector, we employ classical narrowband ray-based mmWave channel model which is given by [30,31]:

(21) $$h_{j}=\;\sum_{i=1}^{P_{j}}G_{j,i}a\left(\theta_{j,i}\right)$$

where $h_{j}$ is the DL channel-vector for *j*th user, that is also required by the BS for precoding and power allocation; $P_{j}$ is the number of resolvable paths from BS to *j*th UE; $G_{j,i}$ is the i.i.d complex-gain of the *i*-th propagation-path of the *j*-th UE having zero mean and unit variance; $\theta_{j,i}$ is the *i*-th path-AoD of the *j*-th UE; $a\left(\theta_{j,i}\right)$ $∊\;{\mathbb{C}}^{Nx1}$ is the steering-vector representing the antenna-response of the *i*-th path of the *j*-th UE.

To obtain $a\left(\theta_{j,i}\right)$, let us suppose that the BS antennas are uniform linear-arrays (ULA), therefore:

(22) $$a\left(\theta_{j,i}\right)=\;{\left[1,\;e^{-j2\pi \frac{d}{\lambda }\sin\left(\theta_{j,i}\right)},\;\ldots ,\;e^{-j2\pi \frac{d}{\lambda }\;\left(M-1\right)\sin\left(\theta_{j,i}\right)}\right]}^{H}$$

where d is the spacing or separation between antenna array elements at the BS; λ Is the wavelength of the carrier frequency.

For the sake of convenience, we write (21) in matrix form as:

(23) $$h_{j}=\;A_{j}G_{j}$$

where $A_{j}=\left[a\left(\theta_{j,1}\right),\;a\left(\theta_{j,2}\right),\;\ldots ,\;a\left(\theta_{j,}P_{j}\right)\right]$ $∊\;{\mathbb{C}}^{NxP_{j}}$; $G_{j}=\;{\left[g_{j,1},\;g_{j,2},\;g_{j,3},\;\ldots ,\;g_{j,P_{j}}\;\right]}^{H}$ $∊\;{\mathbb{C}}^{P_{j}x1}$.

The channel-vectors of all *K*-users are concatenated and represented as follows:

(24) $$H=\left[h_{1},\;h_{2},h_{3},\ldots ,\;h_{K}\right]∊\;{\mathbb{C}}^{NxK}$$

The channel quantization is performed by the codebook:

(25) $${\mathbb{C}}_{j}=\left[c_{j,1,\;}c_{j,2,\;}c_{j,3},\;\ldots ,\;c_{j,2^{b},\;}\right]$$

where $2^{b}$ is the N-dimensional different column-vectors of unit-magnitude, and *b* is the number of bits required for feedback.

The quantization of the *j*th user channel-vector $h_{j}$ to a quantization vector is given by:

(26) $$h_{j}\to c_{j,F_{j\;}}∊\;{\mathbb{C}}^{N\times 1}$$

where $F_{j\;}$ is the quantization index. It is calculated by the following equation:

(27) Fj = sin2i∊{1, 2b}argmin[∠(hj, cj,i, )]= |h˜jHcj,i |2i∊{1, 2b}argmax

where ${\tilde{h}}_{j}$ is the direction of the concerned channel which can be obtained from the following relationship:

(28) $${\tilde{h}}_{j}=\frac{h_{j}}{\left|\left|h_{j}\right|\right|}$$

The quantization vector is transmitted and $F_{j}$ can be feedback from the *j*th UE to BS by employing the feedback bits *b.* When the BS receive such *b*-bits and its associated $F_{j}$, it can produce the feedback channel-vector which is given by:

(29) $${\overset{ˇ}{h}}_{j}=\left|\left|h_{j}\right|\right|c_{F_{j}}$$

The feedback channel-vectors of all *K*-users are concatenated and represented as follows:

(30) $$\overset{ˇ}{H}=\left[{\overset{ˇ}{h}}_{1},\;{\overset{ˇ}{h}}_{2},{\overset{ˇ}{h}}_{3},\ldots ,\;{\overset{ˇ}{h}}_{K}\right]∊\;{\mathbb{C}}^{N\times K}$$

The per-user rate is obtained from (30) using zero-forcing (ZF) linear precoding technique. ZF has the ability to attain near-optimal performance with lower complexity when the BS antennas approach infinity [32]. The signal that is transmitted after ZF precoding is determined by:

(31) $$X=\;\sqrt{\frac{P_{t}}{K}}\;ℾ\;s$$

where $P_{t}$ is the transmit power; $s=\left[s_{1},\;s_{2},s_{3},\ldots ,\;s_{K}\right]$ $∊\;{\mathbb{C}}^{K\times 1}$ is a set of signals dedicated to the *K*-users with unit normalized-power; $ℾ=\left[{ℾ}_{1},\;{ℾ}_{2},{ℾ}_{3},\ldots ,\;{ℾ}_{K}\right]$ $∊\;{\mathbb{C}}^{N\times K}$ is the ZF precoding-matrix which contains *K*-different unit-magnitude precoding-vectors ${ℾ}_{i}$ of order N×1. To obtain the precoding vectors, let us represent the aggregated channel vector as follows:

(32) $$\eta =\;\overset{ˇ}{H}\;{\left({\overset{ˇ}{H}}^{H}\overset{ˇ}{H}\right)}^{-1}$$

Therefore, the precoding-vectors can be obtained as the normalized *i*th column of η as given below:

(33) $${ℾ}_{i}=\;\frac{\eta \left(:,\;i\right)}{\left|\left|\eta \left(:,\;i\right)\right|\right|}$$

The received-signal for the *k*th user can be expressed as:

(34) $$y_{k}=h_{k}^{H}X+n_{k}$$

By putting (31) in (34) we get:

(35) $$=\sqrt{\frac{P_{t}}{K}}h_{k}^{H}\;{ℾ}_{k}s_{k}+\sqrt{\frac{P_{t}}{K}}\overset{K}{\underset{i=1,\;i\neq k}{\sum }}h_{k}^{H}\;{ℾ}_{i}s_{i}+n_{k}$$

where $n_{k}$ is the Gaussian noise-vector at the *k*th user having zero-mean and unit-variance. Therefore, the signal-to-interference noise ratio (*SINR*) at the *k*th user is given by:

(36) $$SINR_{k}=\;\frac{\frac{P_{t}}{K}{\left|h_{k}^{H}\;{ℾ}_{k}\right|}^{2}}{1+\;\frac{P_{t}}{K}\sum_{i=1,\;i\neq k}^{K}{\left|h_{k}^{H}\;{ℾ}_{i}\right|}^{2}}$$

The per-user data rate ℛ is then calculated by:

(37) $$ℛ\;=E\{\log_{2}\left(1+SINR_{k}\right)\}$$

Putting (35) in (36), we get:

(38) $$ℛ\;=E\{\log_{2}\left(1+\frac{\frac{P_{t}}{K}{\left|h_{k}^{H}\;{ℾ}_{k}\right|}^{2}}{1+\;\frac{P_{t}}{K}\sum_{i=1,\;i\neq k}^{K}{\left|h_{k}^{H}\;{ℾ}_{i}\right|}^{2}}\right)\}$$

**Figure 6:**
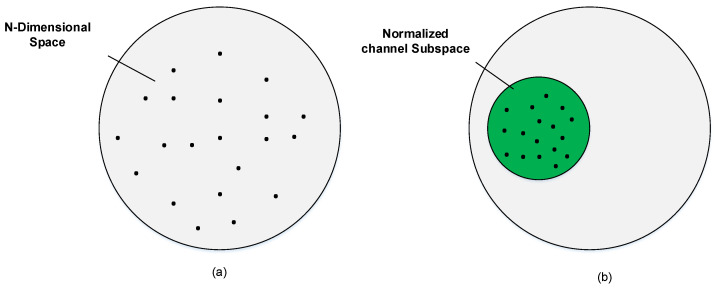
Comparison of the codebooks: (**a**) classical Grassmannian N-dimension; (**b**) Proposed AoD based codebook.

In (38), ℛ is dependent upon ℾ-matrix which in turn depends on the $\overset{ˇ}{H}$-matrix quality. The quantization-vectors are accurately placed on the normalized-channel subspace which is pictorially depicted in Figure 6.

The path-AoD ($\theta_{j,i}$) in (29) is mainly dependent on the nearby hurdles which are around the BS and may not change physically their place in a lot of time as compare to the coherence-time. On the other hand, for the path-gain $\left(G_{j,i}\right)$ a resolvable-path is created by a cluster comprised of scatters which is nearby the *k*th user and it contains a number of unresolvable-paths. Therefore, the *k*th user sees a path-gain that depends on a number of unresolvable-paths. It is clear that the path-gain has much quicker variation than the path-AoDs which vary much slowly. The coherence-time of the angle in which the path-AoDs are considered static and the channel-vector is present in the channel-subspace, is much longer as compare to the channel coherence-time.

Due to joint UL/DL channel estimation benefit, the quantized AoDs can be produced at the BS as well as users. They can also create the steering-matrix. Therefore, the proposed AoD-subspace based codebook is written as:

(39) $$c_{j,i\;}=\;\frac{1}{\sqrt{N}}{\overset{ˇ}{A}}_{j}{\mathbb{Z}}_{i}$$

where, ${\mathbb{Z}}_{i}$ $∊\;{\mathbb{C}}^{k\times 1}$ is the traditional-codebook quantization-vector. The feedback bits *b* is determined as follows:

(40) $$b\;\geq \;\frac{P-1}{3}\;\mathrm{SNR}+\left(P-1\right)\log_{2}\left(\frac{K-1}{c-1}\right)$$

where SNR is computed at the Rx side which is expressed by:

(41) $$\mathrm{SNR}=10\log_{10}\frac{P_{t}}{K}E\left[{\left|\left|h_{k}\right|\right|}^{2}\right]$$

It is clear from (41) that the slope of the *b-*bits (Q−1) is directly proportional to SNR and the rate-gap remains constant. It is also clear that as Q is very much smaller than N, so the proposed AoD-codebook algorithm can substantially reduce the size of codebook and the overhead.

### 3.4. S-CoSaMP Algorithm

**Figure 7:**
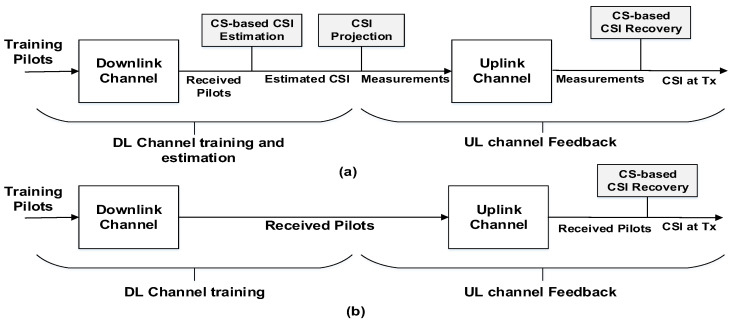
Comparison of the: (**a**) Conventional CS based separate DL and UL channel feedback; (**b**) Proposed CS based joint algorithm for channel estimation.

The conventional UL/DL schemes is a two-step process. In the first step, DL CSI is estimated at the UE and in the second step, the CSI is feedback in UL to BS. These two steps processes are individually optimized which is shown in Figure 7a. To point out the loopholes associated with such technique, we propose a novel approach that performs channel UL and DL feedback jointly. The proposed scheme process is depicted in Figure 7b. In this algorithm, the user feedbacks the pilots to BS without channel-estimation. The BS then extracts the required CIR using the CS algorithm. This is the main advantage of this algorithm as it provides freedom to users from channel-estimation which is a tedious task. Also, the performance of channel-feedback is also enhanced.

The procedure of obtaining the DCIR for UL is the same as explained in subsection A of CS differential CIR. To obtain the received pilots at the BS, we modify (15) as follows:

(42) $${y_{j}}^{\left(k\right)}=\phi \cdot h^{\left(k\right)}+{n_{j}}^{\left(k\right)}$$

We try to use the temporal-correlation of channels by calculating the differential-pilots (D-Pilots) at the BS in two consecutive time-slots *k* − 1 and *k* respectively as [33]:

(43) $$\Delta {y_{j}}^{\left(k\right)}={y_{j}}^{\left(k\right)}-{y_{j}}^{\left(k-1\right)}$$

By putting (42) in (43) we get:

(44) $$=\;\phi \left(h^{\left(k\right)}-h^{\left(k-1\right)}\right)+{n_{j}}^{\left(k\right)}-{n_{j}}^{\left(k-1\right)}\;=\;\phi \Delta h^{\left(k\right)}+\;\Delta {n_{j}}^{\left(k\right)}$$

where $\Delta h^{\left(k\right)}=\;h^{\left(k\right)}-h^{\left(k-1\right)}$, which is the same as (9).

We know that $h^{\left(k\right)}$ and $h^{\left(k-1\right)}$ have sparse structure so the $\Delta h^{\left(k\right)}$ also have sparse nature. So, to reap these sparse benefits, we employ the S-CoSaMP algorithm which has low-complexity and robustness to recover the CIR for LS technique which is given by [34]:

(45) $$\overset{ˇ}{h}=\phi y_{j}$$

This holds true when the CIR has sparse nature. The CoSaMP technique recovers the CIR in the initial time-slot from the pilots received at the BS. It also recovers the DCIR from the D-pilots in the succeeding time-slots. It is also more beneficial than the conventional CoSaMP algorithm as it updates each support of CIR. The conventional CoSaMP does not take into account the sparse structure of CIR. Whereas, the proposed S-CoSaMP utilizes the sparsity inherent in the CIR.

### 3.5. Energy Efficiency Maximization

In this section, we analyze the Uplink energy efficiency (EE) in terms of the number of BSs, the number of users per cell, the density of BS antennas and the pilot reuse factor respectively. The closed form of analytical expressions is derived to provide insights into the energy optimization of small cell based massive MIMO systems. The transmit power of user equipment *i* in *j* cell can be expressed by:

(46) $$P_{ji}=\rho \omega d_{jji}^{\alpha }$$

where ρ is the coefficient of power control. The average transmit power for such UE can then be represented by:

(47) $$E\left\{P_{ji}\right\}=\rho \omega E\left\{d_{jji}^{\alpha }\right\}=\rho \omega \;\frac{ℾ\left(\frac{\alpha }{2}+1\right)}{{\left(\pi \lambda \right)}^{\frac{\alpha }{2}}}$$

Therefore, by using (47), we can calculate the optimized EE for the proposed massive MIMO system by through the statistical power control policy [35].

## 4. Simulation Results

The simulation results are provided to verify our theoretical analysis. Table 1 provides the simulation parameters for the proposed algorithms which are utilized to obtain the simulation results in MATLAB. The sub-space pursuit (SP) technique is used for the recovery process which is more robust to noise and have low complexity [36].

**Figure 8:**
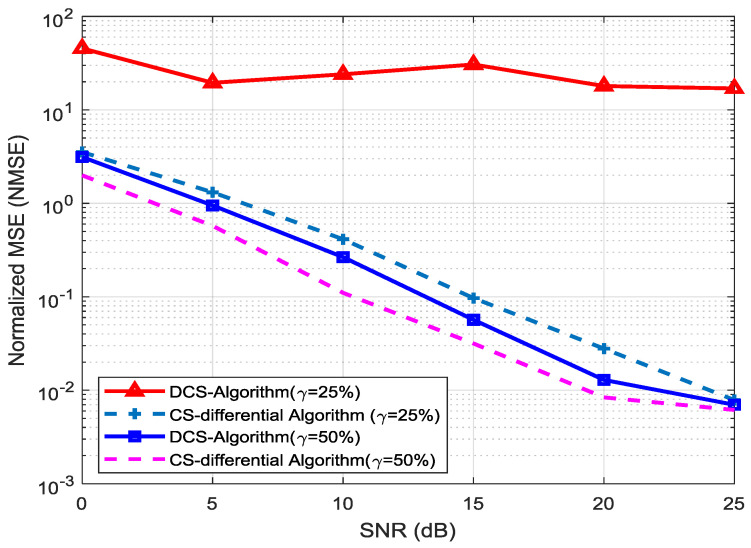
Efficiency analysis of channel-feedback between DCS and CS-differential algorithms.

Figure 8 shows the comparison results for the uplink (UL) channel-feedback for massive MIMO system using the direct compressed-sensing (DCS) and the proposed CS differential techniques. The simulations are performed using two compression ratios (*γ*) for CIRs which are 25% and 50% respectively. The results clearly reveal that the DCS scheme fails for 25% compression ratio as its overhead is lesser than required and the gap between this and the proposed CS differential algorithm gets larger and its NMSE does not reduce as required, while the proposed scheme performs much better than former scheme. For the second compression ratio (50%), the proposed scheme still show better performance as compared to DCS. It also got 6-dB gain over DCS. The proposed CS differential channel feedback algorithm using 25% overhead have similar performance as the DCS algorithm that uses 50% overhead. Its mean that the proposed algorithm decreases the channel-feedback overhead from 50% to 25%, and the net reduction is 25%.

**Figure 9:**
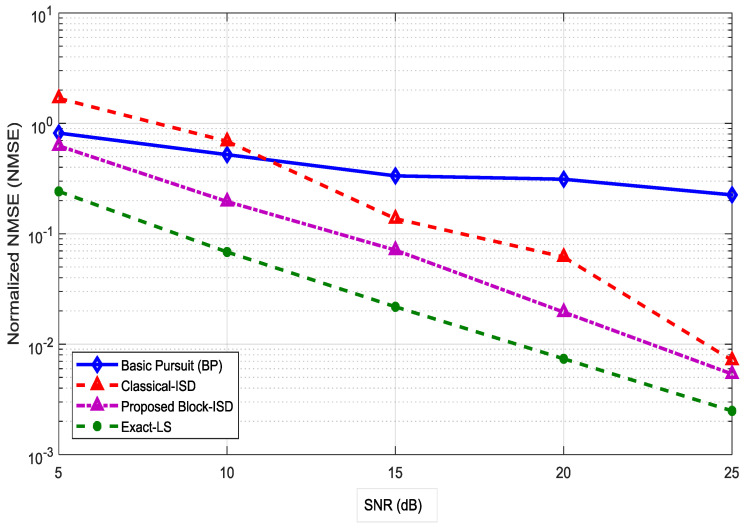
Efficiency analysis of channel-feedback between Block-ISD and classic-ISD algorithms.

Figure 9 shows the NMSE performance comparison of the classical ISD and the proposed Block-ISD algorithms. The results clearly show that the proposed Block-ISD performs better than the classical-ISD and BP algorithms due to more inherent sparsity. The exact LS is a reference for comparison of our results. The number of pilots for LS and other scheme is $p=LN_{t}=32\;\times \;130=4160$ which is far more than the proposed pilots of 640. By taking the percentage between these two pilots signals $\left[\frac{\left(4160-640\right)}{4160}\right]\;\times 100=84.6\%$, it is evident that a significant reduction in the channel-overhead is obtained by using Block-ISD algorithm for channel-estimation of massive MIMO systems.

**Figure 10:**
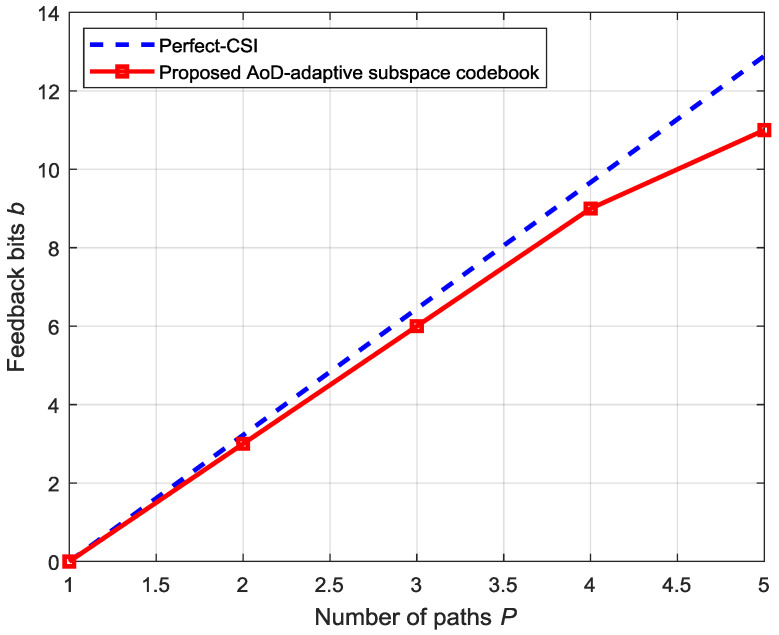
Feedback-bits comparison for maintaining a data-rate gap between the perfect-CSI and proposed AoD-codebook.

Figure 10 illustrates the number of feedback bits *b* required to maintain a constant rate-gap between the ideal CSI and the proposed practical AoD-adaptive subspace codebook. We can see that the rate gap is limited to within 0.12 bps/Hz. The graph is linear, therefore, the number of feedback bits required is linearly proportional to the number of paths. By comparing this simulation result with our theoretical formulation in (39), it is clearly validating it.

**Figure 11:**
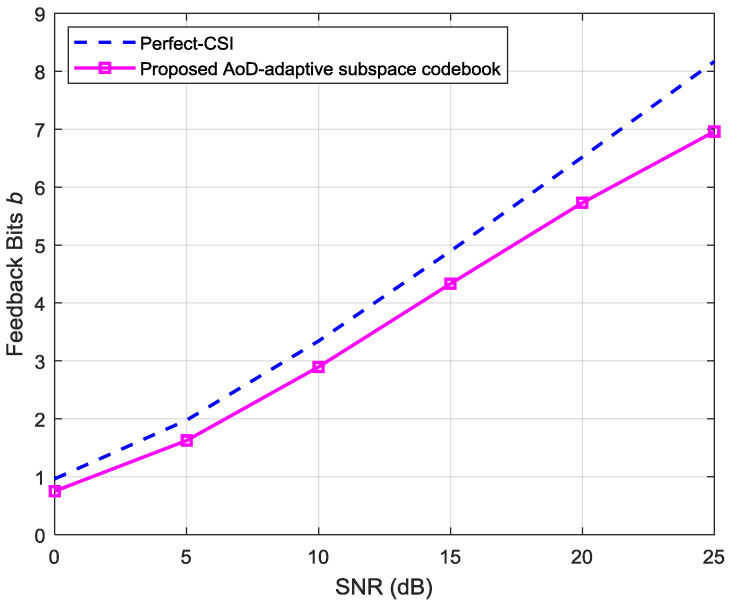
Comparison of the required number of feedback-bits with increasing the SNR between the ideal CSI and the proposed AoD-codebook schemes.

Figure 11 compares the required feedback bits versus the SNR between the ideal scenario of perfect-CSI and the proposed scheme with limited channel-feedback. We observe that the required number of feedback bits vary linearly with the SNR under consideration.

**Figure 12:**
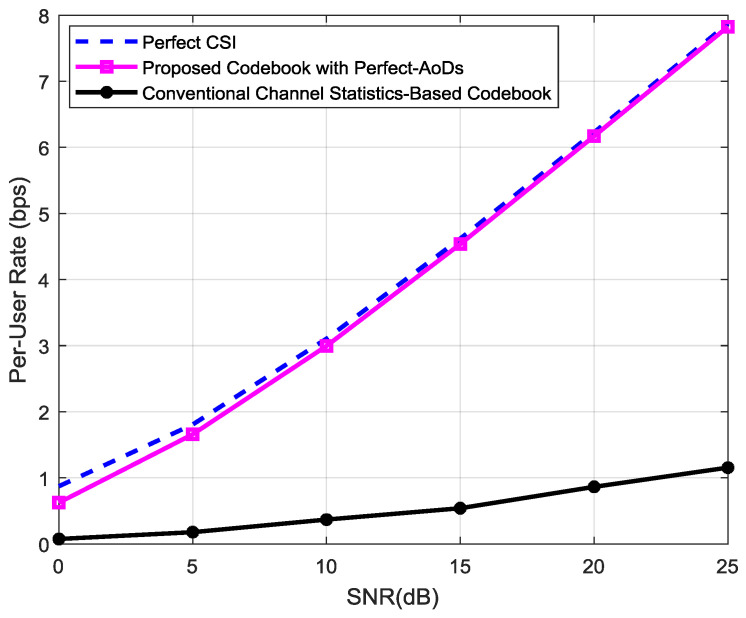
Comparison of the per-user rate of the ideal perfect-CSI and the proposed scheme with quantized-channel feedback in terms of SNR.

Figure 12 shows the per-user data rate comparison of the ideal condition, conventional channel statistics-based codebook and the proposed AoD-adaptive subspace codebook with perfect path-AoDs. The feedback bits *b* is constant for the quantized channel feedback algorithms. It is clear from the results that the per-user rate gap between the ideal perfect-CSI and the proposed AoD-codebook is at a constant level when the Rx SNR increases. From the Figure, the rate gap at 0 dB SNR is approximately 0.18 bps/Hz, whereas, at 15 dB SNR, the proposed AoD-codebook overlaps on the ideal case. This validates our theoretical analysis. The rate gap between the ideal CSI and the conventional-codebook increases with increasing the SNR, which is undesired in practice. Also, the proposed AoD-codebook performs much better than the conventional-codebook.

**Figure 13:**
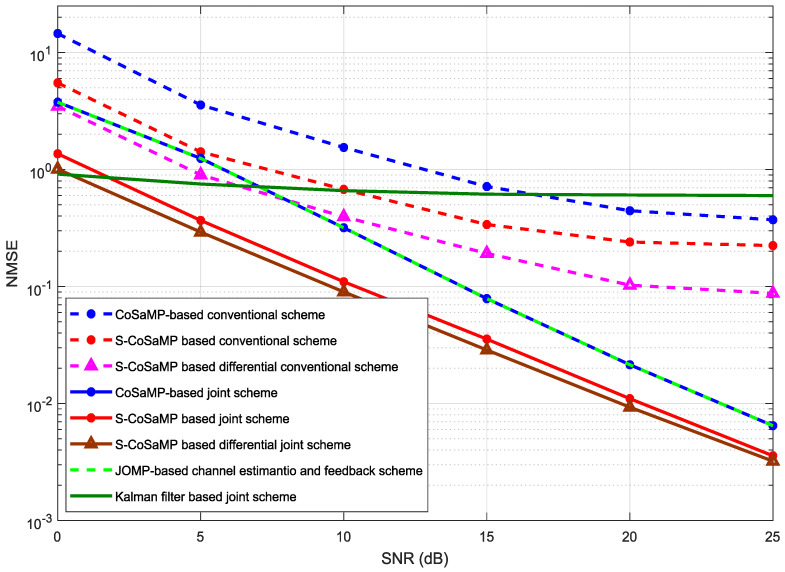
Comparison of the proposed and the conventional channel feedback algorithms in terms of NMSE versus SNR.

Figure 13 shows the NMSE comparison of the proposed S-CoSaMP and the conventional algorithms. The conventional algorithms are represented by dotted-lines while the joint algorithms are represented by solid lines. It is clear from the Figure that the S-CoSaMP based differential joint scheme performs best as compare to other algorithms as it deploys temporal-correlation and sparsity. The proposed algorithm has the least NMSE which means reduced feedback-overhead as compared to the conventional schemes. On the second best is the S-CoSaMP joint algorithm which performs better than the conventional schemes. The JOMP and CoSaMP joint schemes have the same performance as their common support vectors are not satisfied. The Kalman Filter response is not better as it requires the autocorrelation information of the channel. We also observe that the NMSE gap between the conventional algorithms and the proposed algorithms increases with increasing SNR. Thus, it also indicates the dominance of our proposed algorithms. 

**Figure 14:**
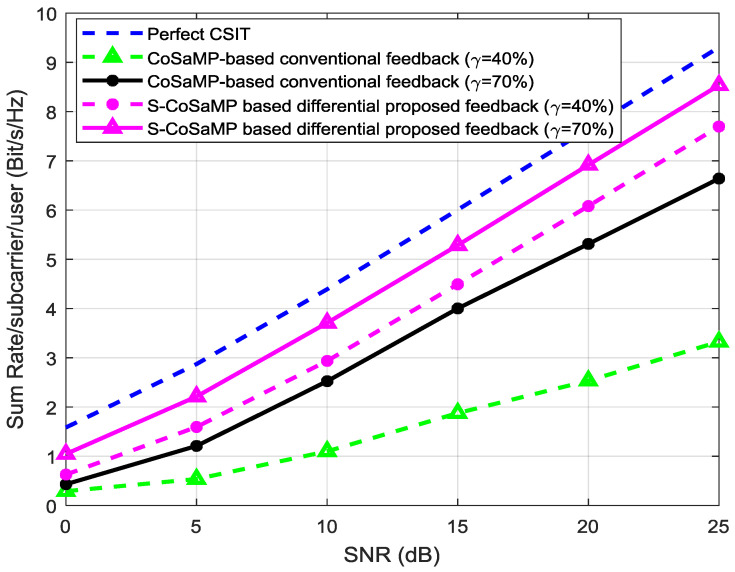
Comparison of sum-rate between the ideal CSI; proposed S-CoSaMP and the conventional CoSaMP algorithms under two-compression ratios.

Figure 14 compares the sum-rate between the ideal perfect-CSI, S-CoSaMP, and the conventional CoSaMP algorithms. We consider two compression ratios (*γ*) for comparing their performance evaluation. The perfect-CSI case is used for reference purpose with which we compare our proposed results. It is clear from the Figure that our proposed S-CoSaMP based joint differential feedback shows better performance and it is operating near the ideal case of perfect-CSI at *γ* = 70%. The proposed S-CoSaMP at *γ* = 40% perform better than the conventional CoSaMP which is operating with the overhead of *γ* = 70% and they have approximately the same data-rate. The conventional CoSaMP fails at the overhead of *γ* = 40% because the rate gap between this and the ideal CSI gets much larger which is undesired in practice. It shows that our proposed algorithm provides 30% reduction in overhead and improves the channel feedback efficiency as compared to the conventional algorithm in attaining the same sum-rate.

**Figure 15:**
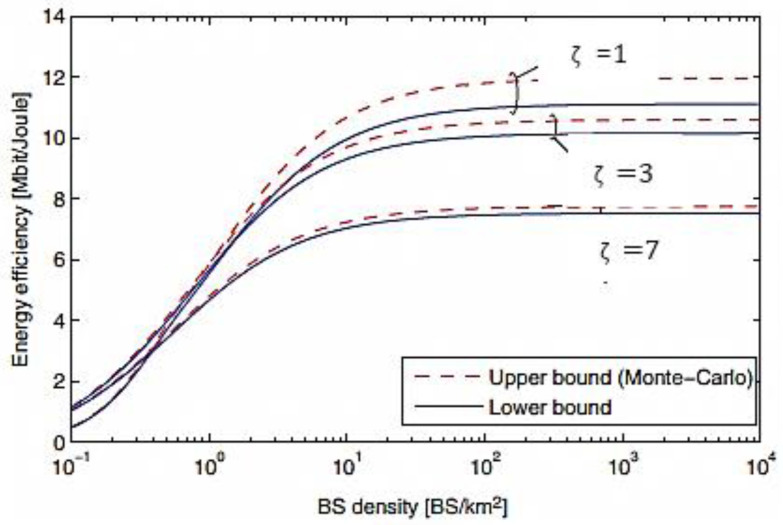
Energy Efficiency versus the BS density comparison of massive MIMO system for different SINRs (ζ).

Figure 15 shows the energy efficiency of the proposed massive MIMO system against the density of BSs. It is clear from the Figure that the EE gap between the upper and lower bounds for all three signal-to-interference ratios (SINRs) ζ is very small and it also decreases as SINR increases. The analytical expression of the SINR is given by:

(48) $$\zeta =\;\frac{{\left(1-\epsilon^{2}\right)}^{2}}{\left(1-\epsilon^{2}\right)\left(\frac{1}{\beta \left(\partial -1\right)}+\epsilon^{2}\right)}$$

where ϵ is the level of hardware impairment, β is the optimization variable and ∂ is the power level coefficient respectively. Moreover, the EE is significantly improved with increasing the BS density. This means that by deploying smalls cells, the EE greatly improves which makes it an attractive solution to such issue. Furthermore, the EE decreases as the SINR increases which make a constraint on the EE and for this purpose, a target SINR should be specified from a practical perspective to obtain the required EE.

**Figure 16:**
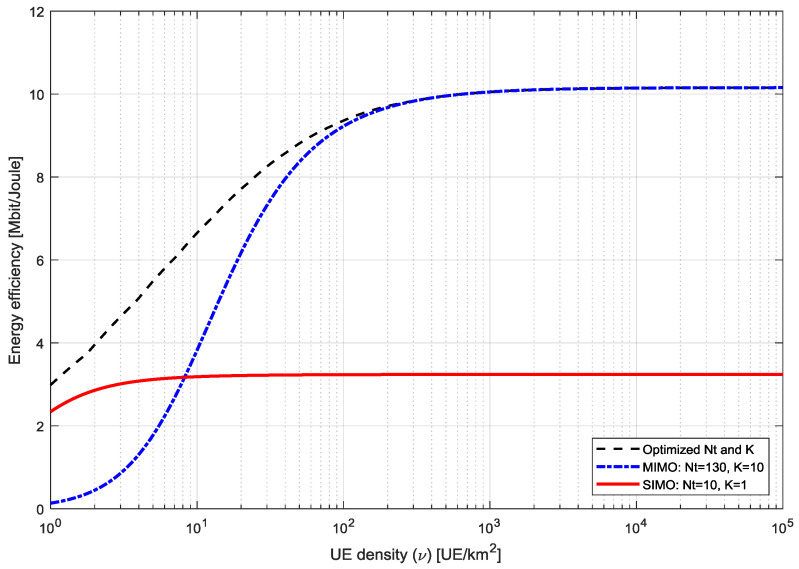
Energy Efficiency versus the UE density comparison of massive MIMO system.

Figure 16 illustrates the EE against the UE density for the average SINR (ζ) of 3 dB respectively. It is compared with two references usage cases, such as single-input multiple outputs (SIMO) and MIMO for performance evaluation. It is clear from the results that the EE becomes independent as the UE density increases to a large level. We can see that the EE saturation levels occurs at UE density of 100 and above respectively for the optimized and MIMO cases. The saturation for SIMO case occurs at UE of 2 and above. Moreover, we can observe that the proposed system shows optimized performance than the other two competing cases which makes it suitable for practical usage scenarios of small cells dense networks.

**Figure 17:**
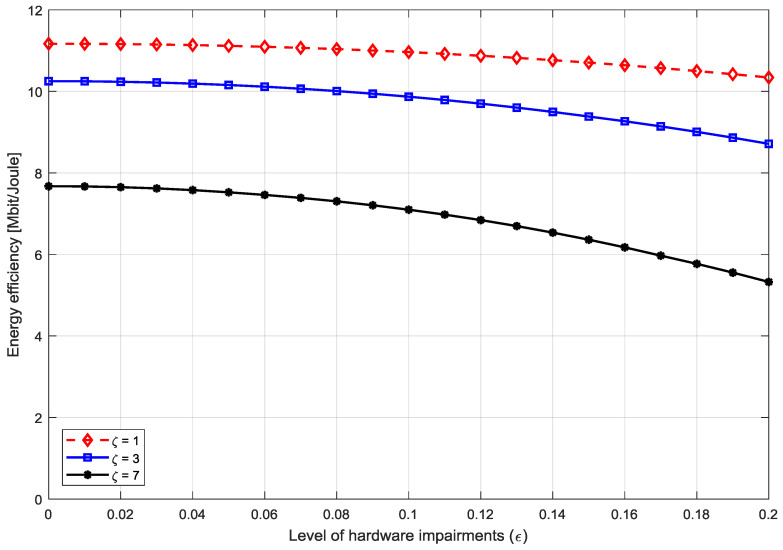
Energy Efficiency versus the level of hardware impairment of massive MIMO system for different SINRs.

Figure 17 shows the EE levels against the level of hardware impairments for three different SINR levels. It is clear from the results that the EE decreases with increasing hardware impairments. It is due to the decay of the desired signal power.

The loss can be high when the SINR increases to a large level which means that the hardware impairment substantially affects the EE in high SINR levels. Moreover, the EE loss is negligible for hardware impairment of level of 0.1 and below. Therefore, it can be concluded that for the moderate hardware impairment level, the effect on the system under consideration is negligible.

## 5. Conclusions

This paper scrutinizes the feedback and pilot overhead issues of uplink and downlink channel estimation in massive MIMO systems. It also investigates the EE evaluation in terms of BS density, UE density, and hardware impairment levels respectively. For the UL-channel estimation, it is concluded that by using temporal-correlation of massive MIMO time-varying channels which produces the compression-efficient differential CIR has 25% less overhead requirement or in other words, 25% more effective as compare to the direct CS (DCS) algorithm. It is also shown that the proposed CS algorithm has improved NMSE than the DCS algorithm. For the DL-channel estimation, the proposed Block-ISD scheme shows enhanced performance as compare to classical-ISD and BP schemes. It utilizes spatial-correlation of massive MIMO channels which produces block-sparse CIR that have more compression-efficiency and due to such mechanism, the pilot-overhead is decreased by 84.6%. In other words, the efficiency of the Block-ISD is 84.6% more than the classical-ISD or BP techniques. The AoD-adaptive subspace codebook provides a substantial reduction in the training and feedback overhead as well as the codebook size which results in improved data-rate and bandwidth-efficiency. It uses the idea that the time-based variation in channels path-AoDs is much slower than path-gains. The proposed results are so close to the theoretical analysis in terms of per-user data-rate and feedback bits. As we have proved that the feedback bits vary linearly with the number of paths *P* rather than the whole N-dimensional space which provides freedom from the large BS antenna-based feedback bits scaling. The S-CoSaMP algorithm is used for joint channel training and feedback which is based on CS that further reduces the channel overhead. It has reduced the joint UL/DL overhead by 30%. It also provides the benefit of getting rid of the burden on the UE which estimate their channel and then feedback to BS. The UE directly feedback their pilots to BS and then the CS algorithm obtain the required CSI at the BS. The EE levels of the proposed system outperform the SIMO and MIMO usage cases which makes it suitable for small cells dense networks operation. Also, the EE levels are optimum in upper and lower bounds which are defined using three different SINR level of 1, 3 and 7, respectively. The EE gap between both bounds is close enough which decreases further with increasing SINR levels. We also conclude that the EE level decreases as the SINR levels increases which provide information to specify a target SINR for each considered usage case in practical perspective. Therefore, the EE increases with increasing BS density, UE density, and lower hardware impairment levels respectively. Our future work is focused on reducing the channel overhead for which we will utilize the spatial-correlation of CSI from various UEs at the BS. Also, the quantization error of AoD in terms of feedback bits will be evaluated and analyzed.

## Figures and Tables

**Table 1:** Proposed System Simulation Parameters.

S. No	Parameter	Symbol	Value
1	Max. Channel Length	*L*	32
2	Pilot length	p	640
3	OFDM symbol-length	S	2048
4	Signal-to-Noise-Ratio	SNR	25 dB
5	Block Elements	*B*	8
6	Noise Variance	$\sigma_{n}$	1
7	Transition-Probability	*P*_01_	0.05 (5%)
8	Channel-Sparsity	*μ*	0.1 (10%)
9	Doppler-frequency	*f_d_*	10 Hz
10	Slot-interval	*τ*	1 ms
11	Channel-variance	$\sigma_{w}$	1
12	Correlation coefficient	ρ	0.0628
13	Number of BS antennas	*N*_t_	130
14	Number of User antennas	*K*	10
15	Number of Paths	*P*	5
16	AoD Feedback bits	*b*	5
17	Sensing-Matrix	*φ*	Random Gaussian
18	Static Power Consumption	*P_s_*	10 W
19	Circuit power per antenna	*P_ca_*	0.1 W
20	Path Loss Exponent	*α*	3.76
21	Coherence Block Length	*S*	400
22	Coherence Time	*T_c_*	2.16 ms
23	Bandwidth	BW	20 MHz
24	Macrocell Radius	r	250 m
